# Emergent magnetic monopole dynamics in macroscopically degenerate artificial spin ice

**DOI:** 10.1126/sciadv.aav6380

**Published:** 2019-02-08

**Authors:** Alan Farhan, Michael Saccone, Charlotte F. Petersen, Scott Dhuey, Rajesh V. Chopdekar, Yen-Lin Huang, Noah Kent, Zuhuang Chen, Mikko J. Alava, Thomas Lippert, Andreas Scholl, Sebastiaan van Dijken

**Affiliations:** 1 Advanced Light Source, Lawrence Berkeley National Laboratory (LBNL), 1 Cyclotron Road, Berkeley, CA 94720, USA.; 2 Laboratory for Multiscale Materials Experiments (LMX), Paul Scherrer Institute, 5232 Villigen, Switzerland.; 3 Physics Department, University of California, Santa Cruz, 1156 High Street, Santa Cruz, CA 95064, USA.; 4 COMP Centre of Excellence, Department of Applied Physics, Aalto University, P.O. Box 11100, FI-00076 Aalto, Espoo, Finland.; 5 Institut für Theoretische Physik, Universität Innsbruck, Technikerstraße 21A, A-6020 Innsbruck, Austria.; 6 Molecular Foundry, LBNL, 1 Cyclotron Road, Berkeley, CA 94720, USA.; 7 Department of Materials Science and Engineering, University of California, Berkeley, Berkeley, CA 94720, USA.; 8 Materials Sciences Division, LBNL, 1 Cyclotron Road, Berkeley, CA 94720, USA.; 9 School of Materials Science and Engineering, Harbin Institute of Technology, Shenzhen, Guangdong 518055, China.; 10 Department of Chemistry and Applied Biosciences, Laboratory of Inorganic Chemistry, ETH Zurich, Switzerland.; 11 NanoSpin, Department of Applied Physics, Aalto University School of Science, P.O. Box 15100, FI-00076 Aalto, Finland.

## Abstract

Magnetic monopoles, proposed as elementary particles that act as isolated magnetic south and north poles, have long attracted research interest as magnetic analogs to electric charge. In solid-state physics, a classical analog to these elusive particles has emerged as topological excitations within pyrochlore spin ice systems. We present the first real-time imaging of emergent magnetic monopole motion in a macroscopically degenerate artificial spin ice system consisting of thermally activated Ising-type nanomagnets lithographically arranged onto a pre-etched silicon substrate. A real-space characterization of emergent magnetic monopoles within the framework of Debye-Hückel theory is performed, providing visual evidence that these topological defects act like a plasma of Coulomb-type magnetic charges. In contrast to vertex defects in a purely two-dimensional artificial square ice, magnetic monopoles are free to evolve within a divergence-free vacuum, a magnetic Coulomb phase, for which features in the form of pinch-point singularities in magnetic structure factors are observed.

## Teaser

We present direct, real-time observations of emergent magnetic monopole dynamics in highly frustrated artificial spin ice.

## INTRODUCTION

Spin ices (*1*–*3*) represent a class of geometrically frustrated magnetic materials that, at low temperatures, enter a phase that is strongly dominated by short-range moment correlations and the absence of long-range order (*3*). They are composed of corner-sharing tetrahedra, where the rare-earth ion moments occupy the corners of these tetrahedra. Local constraints force these moments to obey the so-called ice rules (*2*, *4*) of two moments pointing in and two moments pointing out of each tetrahedron. With the concept of emergent magnetic charges (*5*), where each dipole moment is replaced by a dimer of two opposite magnetic charges, configurations obeying the ice rule can be mapped onto a divergence-free field, a so-called Coulomb phase (*6*, *7*), which acts like a vacuum for local excitations that act as emergent magnetic monopoles (*5*).

**Fig. 1:**
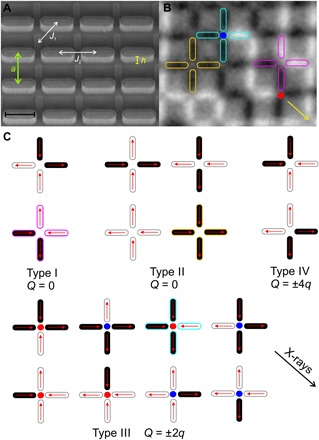
Thermally activated two-dimensional artificial square ice with height offsets between nanomagnets. (**A**) Tilted-sample scanning electron microscopy (SEM) image of an artificial square ice with an introduced height offset *h*, which can be varied from sample to sample, until the competing interactions *J*_1_ and *J*_2_ are equalized and an extensive spin ice degeneracy is achieved. Scale bar, 400 nm. (**B**) XMCD image of the same artificial square ice array. Nanomagnets with moments pointing toward the incoming x-rays (indicated by a yellow arrow) appear dark, while those opposing the x-ray direction appear with bright contrast. (**C**) The 16 possible moment configurations on a four-nanomagnet vertex are traditionally listed into four topological types. Without a height offset (*h* = 0 nm), the ice rule–obeying (two-in-two-out) type I and II configurations have a significantly different energy. Once a critical height offset is introduced, their energies are equalized and spin ice degeneracy is realized. Highlighted with magenta, cyan blue, and yellow frames in (B) and (C) are type I, type II, and type III vertices, respectively.

Two-dimensional artificial square spin ice (*8*) was initially introduced to mimic these ice rule constraints, with the attractive prospect of directly visualizing the consequence of geometrical frustration using appropriate imaging techniques (*8*, *9*). However, it has been shown to lack typical spin ice degeneracy and residual entropy, mainly due to nonequivalent nearest-neighbor distances of nanomagnets meeting at four-nanomagnet vertices (*8*, *10*). Simple thermal annealing procedures have been shown to lead artificial square ice to access long-range ordered ground-state configurations (*9*, *11*, *12*). The introduction of height offsets between the two sublattices of the square geometry has long been proposed as means to restore spin ice degeneracy by equalizing the relevant vertex interactions (*J*_1_ and *J*_2_ in Fig. 1A and fig. S1) (*10*), but a first experimental realization of such square ice systems could only be most recently achieved (*13*). An extensive degeneracy was achieved, accessing the aforementioned Coulomb phase and emergent magnetic monopoles that arise mostly in the form of ice rule breaking type III vertex defects (Fig. 1, B and C). These monopole defects result in an overall net magnetic charge *Q* = ±2*q* residing at the corresponding four-nanomagnet vertex site (Fig. 1C). In contrast to that, ice rule–obeying moment configurations (types I and II) result in neutral *Q* = 0 vertex sites. So far, the patterned nanomagnets had blocking temperatures far above room temperature, making thermal annealing impractical and direct observations of the real-time thermodynamics of emergent magnetic monopoles impossible. Therefore, in analogy to previous work on athermal two-dimensional artificial spin ices (*8*, *14*, *15*), a demagnetization protocol was used to access quasi-frozen low-energy states in the patterned square ice arrays (*13*). Alternatively, researchers have sought Coulombic behavior in the highly frustrated artificial Kagome spin ice (*16*). Although it does feature some analogies to pyrochlore spin ice (*17*, *18*), the thermodynamics of emergent magnetic charge defects in this lattice have been shown to be strongly confined in nature (*19*), which is typical for all known two-dimensional artificial spin ice systems (*9*, *20*). The introduction of XY-mesospins within four-nanomagnet vertices (*21*) allows a two-dimensional square ice system to access a spin liquid-type phase. However, it is not clear how one can accurately account for emergent magnetic monopoles when taking XY-mesospins that reside at the vertices into account. Thus, the gap toward a direct comparison to both theoretical and experimental studies on the statistical physics of pyrochlore spin ice and emergent magnetic monopoles is not overcome until an artificial square ice system with variable height offsets (*13*) is realized that exhibits moment fluctuations, ideally at experimentally accessible temperatures (*20*, *22*). In addition, these fluctuations are ideally visualized with an appropriate imaging technique.

Here, we present two-dimensional artificial square ice patterns consisting of nanomagnets with variable height offsets and thermally induced moment reorientations at experimentally accessible temperatures. This quasi–three-dimensional lattice is realized by placing nanomagnets with lengths *L* = 400 nm, widths *W* = 100 nm, and thicknesses *d* = 3 nm onto a square lattice with lattice parameter *a* = 550 nm on top of a pre-etched silicon (100) substrate (Fig. 1A). While one set of nanomagnets (Fig. 1A and orange frame in fig. S1) is grown on the base of the pre-etched substrate, the second set (Fig. 1A and blue frame in fig. S1) is grown on top of plateaus whose height can be accurately controlled. Magnetic configurations and thermal fluctuations are then directly visualized using synchrotron-based photoemission electron microscopy (PEEM) (*23*), using x-ray magnetic circular dichroism (XMCD; Fig. 1B) (*24*), which gives a direct measure of the magnetization direction of each individual nanomagnet. More details on sample fabrication and characterization are provided in the Materials and Methods section, in addition to a schematic overview in fig. S2.

## RESULTS

### Thermal annealing

**Fig. 2:**
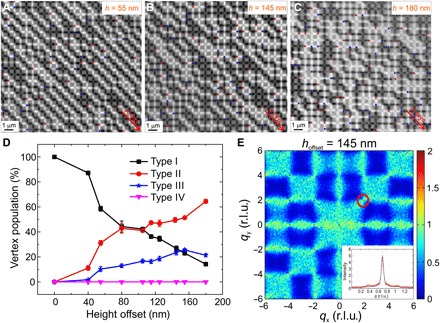
Vertex populations, magnetic structure factors, and pinch-point analysis. (**A** to **C**) Low-energy moment configurations achieved after thermal annealing in artificial square ice arrays with height offsets of (A) *h* = 55 nm, (B) *h* = 145 nm, and (C) *h* = 180 nm. Scale bars, 1 μm. (**D**) Average vertex-type populations of thermalized artificial square ice, plotted as a function of introduced height offsets. Type I vertices dominate the configuration landscape up to an offset of 40 nm but continue to decrease in population with increasing height offset. A turning point is observed at an offset around 80 nm, where type I and II populations reach nearly identical values. The type II population continues to rise with increasing height offset and reaches twice the population of type I vertices at a height offset between 145 and 155 nm. As the height offset is increased beyond this critical value, type II vertices start to fully dominate the moment configuration in the spin ice. (**E**) Magnetic structure factor of an artificial square ice with a height offset of 145 nm. The structure factor is calculated from magnetic moment configurations recorded with PEEM imaging and exhibits pinch-point singularities, a typical feature of a magnetic Coulomb phase. The line scan through (*q*_*x*_, *q*_*y*_) = (2, 2) is fitted by a Lorentzian function (black curve in inset) from which an average spin-spin correlation length ξ = 10.8*a* ± 0.1 is derived. r.l.u., reciprocal lattice unit.

As a first step, we apply a thermal annealing protocol on our artificial square ice structures with various height offsets. Defining the blocking temperature *T*_B_ as the temperature, where moment reorientations of the individual nanomagnets occur at the time scale needed to obtain a single XMCD image (7 to 10 s per image) (*9*, *20*), the patterned structures had blocking temperatures around 330 K. Therefore, the sample is heated to 390 K, with a waiting time of 100 min, before cooling down below the blocking point to 300 K. The achieved moment configurations are then imaged (Fig. 2, A to C) and first analyzed in terms of vertex-type populations (Fig. 2D). The results reveal a transition from a long-range ordered (type I) ground state (*9*) to increasingly disordered configurations with an increasing number of type III vertex defects. Furthermore, square ice systems with height offsets of 145 to 155 nm feature twice as many type II as type I vertices, indicating the restoration of spin ice degeneracy within this critical height offset regime. This is further confirmed by the average magnetic structure factor of an artificial square ice array with a height offset of 145 nm (Fig. 2E). The data reveal that the system accessed a phase that features properties of a cooperative paramagnet, exhibiting pinch-point singularities in the magnetic structure factor, indicative of algebraically decaying correlations resulting from the local ice rule (*7*). That is, an effective Coulomb phase (*7*) is accessed with topological defects (type III vertices) that can be described as emergent magnetic monopoles (*5*, *7*, *13*). The locations of the pinch points are determined by the symmetry of the lattice. Analyzing one pinch point singularity in detail [we choose the point (*q*_*x*_ = 2, *q*_*y*_ = 2) for consistency with previous experiments (*13*)], we find an intensity distribution that fits well into a sharp Lorentzian curve (Fig. 2E) from which a spin-spin correlation length (*13*) of 10.8*a* ± 0.1 is obtained (see Materials and Methods). The finite width of our pinch points is a result of both the finite size of the lattice and disruptions of ice rule ordering by topological defects. The correlation length calculated from the width is related to the average ice rule–obeying string length connecting emergent magnetic monopole defects. Magnetic structure factors as a function of introduced height offsets are shown in fig. S3. As we increase the height offset between nanomagnets beyond the critical regime of 145 to 155 nm, we observe a transition toward phases featuring multidomain type II vertex patterns (Fig. 2C and fig. S3F).

### Real-time thermodynamics

**Fig. 3:**
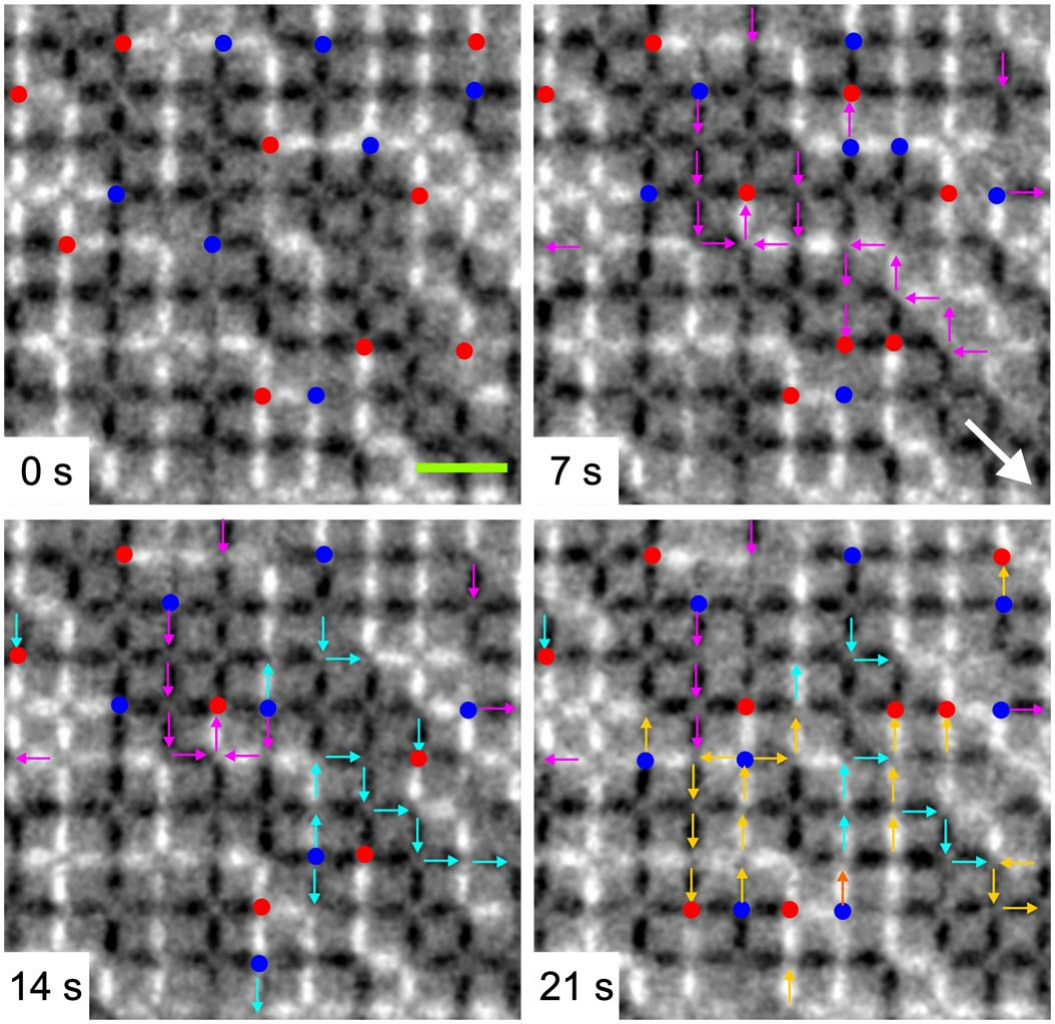
Temporal evolution of emergent magnetic monopoles. XMCD image sequence (recorded at *T* = 190 K) highlighting the thermally driven motion of emergent magnetic monopole defects (blue dots: *Q* = −2*q*, red dots: *Q* = +2*q*) in two-dimensional artificial square ice with a height offset *h* = 145 nm. Arrows of different colors (magenta, cyan blue, and yellow) indicate sequential changes in moment configurations at each instant of time (7, 14, and 21 s). The green bar and the big white arrow indicate a length of 1 μm and the incoming x-ray direction, respectively.

In pyrochlore spin ice, emergent magnetic monopoles are predicted to behave as classical magnetic analogs to electric charges with a Coulomb-type interaction (*5*). Evidence regarding their existence and behavior has relied heavily on scattering or macroscopic measurement techniques (*25*, *26*), while the macroscopically degenerate artificial square ice discussed here offers the unique opportunity to shed light into the dynamic behavior of emergent magnetic monopole defects via real-space imaging. For this, we fabricated a second sample with a height offset of 145 nm and a blocking temperature of 160 K consisting of nanomagnets with length *L* = 400 nm, width *W* = 100 nm, and thickness *d* = 2.5 nm. Obtaining XMCD image sequences (7 to 10 s per image) at various temperatures between 160 and 210 K, we are able to directly visualize real-time thermal fluctuations and motion of emergent magnetic monopoles (see Fig. 3 and movies S1 and S2) and characterize their temperature-dependent behavior. In Fig. 3, a short sequence of XMCD images recorded at 190 K is shown, with a time frame of 14 s separating them. Following sequential changes in the XMCD contrast, when going from frame to frame (marked by correspondingly colored arrows in each frame of Fig. 3), we are able to track the motion of emergent magnetic monopoles as a function of time. We find that the motion of magnetic monopoles is free in all possible directions, with the only limitation being that certain motion steps are unlikely, as they would require the generation of type IV (*Q* = ±4*q*) defects, which is energetically unfavorable and never detected within all our observations. This “free” motion of emergent magnetic monopoles in a two-dimensional lattice with a critical height offset between nanomagnets stands in contrast to the purely two-dimensional artificial square ice (*h* = 0 nm), where magnetic monopoles are highly confined within domain boundaries separating type I domains. As a consequence of this restricted motion, the concept of freely moving Coulomb-type magnetic monopoles becomes highly questionable in purely two-dimensional spin ice systems (*9*, *27*).

### Debye-Hückel theory and monopole crystallization

**Fig. 4:**
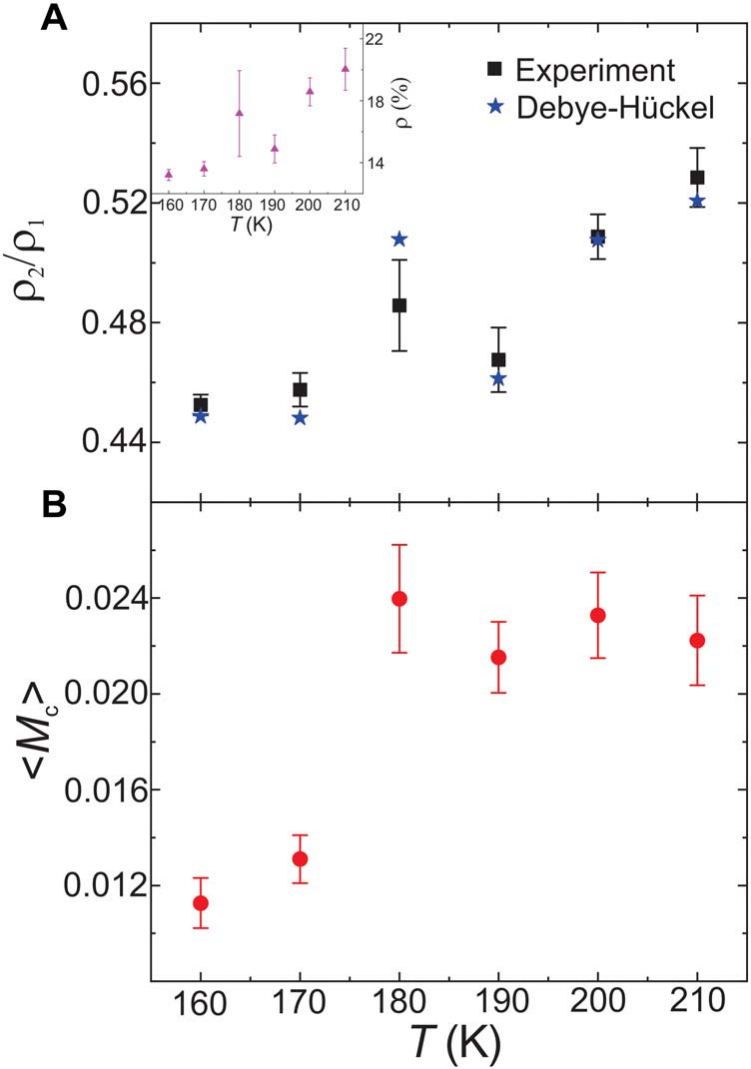
Debye-Hückel behavior and crystallization of emergent magnetic monopoles. (**A**) Ratio of correlated to uncorrelated monopole defects observed in the *h* = 145 nm sample (black dots from Eq. 2) compared to the prediction from the Debye-Hückel theory with Bjerrum association corrections (blue stars from Eq. 1). The error bars correspond to real-time thermal fluctuations over observations of approximately 15 min at each temperature. The best fit is obtained for a magnetic charge *Q* = 9.765 × 10^−12^Am and a magnetization *M* = 54 kA/m in the Debye-Hückel analysis. The overall monopole density ρ as a function of temperature is shown as an inset. (**B**) Crystallization order parameter over the same temperature range.

A collection of free charges should act according to the predictions of a plasma theory. These theories typically account for a process that dissociates dipoles into monopoles, much like electrolytes dissolve in a solution. Commonly used to model electrolyte and plasma systems, the Debye-Hückel theory (*28*) describes a plasma in which charge pairs may spontaneously enter the system and separate through Bjerrum-ion dissociation (*29*). While the Debye-Hückel theory was successfully applied in modeling the dynamics of emergent magnetic monopoles in pyrochlore spin ice (*18*, *30*), it is only with a thermally activated and macroscopically degenerate artificial square ice realized in this work that a direct visual interpretation of magnetic monopole motion within the framework of the Debye-Hückel theory can be delivered. Using this theory, we view the square ice lattice as a plasma of emergent magnetic charges. Charge populations (see inset in Fig. 4A for the overall monopole density plotted as a function of temperature) are divided into correlated and uncorrelated magnetic monopoles. Correlated charges are uniformly distributed in pairs, while uncorrelated charges are simply uniformly distributed (fig. S4). Analyzing the aforementioned XMCD sequences, we extract both the densities of correlated (ρ_2_) and uncorrelated (ρ_1_) magnetic monopoles (see Material and Methods) and plot the ratio ρ2ρ1 as a function of temperature (black dots in Fig. 4A calculated from Eq. 2 in Materials and Methods). This experimentally derived temperature dependence is in good agreement with the theoretical prediction (blue stars in Fig. 4A calculated from Eq. 1 in Materials and Methods), implying Coulombic-type interactions between the emergent magnetic monopoles. The agreement is achieved for an emergent magnetic monopole charge of *Q* = 9.765 × 10^− 12^Am, which corresponds to a saturation magnetization of the permalloy nanomagnets of *M*_S_ ≈ 54 kA/m. This value is substantially lower than the saturation magnetization for bulk permalloy, but it is not far off from 85 kA/m reported for patterned 3.2-nm-thick permalloy kagome structures (*31*). A similar reduction in the saturation magnetization has been reported on patterned FePd thin films (*32*), which indicates that the apparent decrease in *M*_S_ can be attributed to smaller activation volumes that initiate moment reversals, once thermal effects gain significance in these nanostructured thin films.

The Bjerrum association approach assumes charges to be either correlated or uncorrelated. However, images of charge distributions reveal that charges also exist in more complicated states (*33*). For example, charges may slightly correlate by aligning with next-next nearest neighbors, lie adjacent to two or more charges, or any other combinatoric possibilities. To investigate the extent of charge ordering, a crystallization order parameter (see Materials and Methods) (*34*) is calculated and plotted versus temperature in Fig. 4B. Because this parameter remains well below 1, we can conclude that the global ordering needed for crystallization is absent. The parameter increases to an asymptotic value at higher temperatures due to crowding of magnetic charges into cohabitating neighboring sites. The jump toward this asymptote occurs around 180 K, the temperature associated with the spike in the charge density ratio. Both total monopole density (Fig. 4A, inset) and the ρ2ρ1 ratio (Fig. 4A) show a sudden rise at 180 K. Barring any complex, nonmonotonic behavior missed by theoretical studies and previous experiments, this jump in total monopole density is likely a result of the limited field of view of approximately 17 μm. The large SD in this measurement corroborates this. The ρ2ρ1 ratio implicitly depends on total charge density, adding an outlier within otherwise monotonic temperature dependencies. The lack of monopole crystallization in our quasi–three-dimensional artificial square ice stands in contrast to charge crystallites observed in two-dimensional artificial kagome spin ice (*12*). This can be attributed to the chemical potential in this type of system being well above the critical chemical potential (*33*) of μ_*c*_ = 0.80777 under which charge crystallization can be expected.

## DISCUSSION

Although the finite-temperature blocking temperature of the patterned nanomagnets prevents measurements of dynamics at even lower temperatures, a comparison of chemical potential to crystallization energy leads us to conclude that this system supports a spin ice ground state. A system of magnetic charges on the pyrochlore lattice with a tunable chemical potential is expected to form low temperature arrangements of alternating positive and negative charges (fig. S5) when the chemical potential is smaller than a critical value (*34*). By basic energetic considerations [see (*34*) and Materials and Methods], the critical chemical potential is half the Madelung constant of the lattice in question. Alternatively, if the chemical potential is high compared to the energy required to ionize a pair of charges, then the pair will separate at temperatures where charges form in the system. Although the square ice chemical potential is less than that of the pyrochlore lattice, it is still comfortably higher than the critical value (see Materials and Methods), coinciding perfectly with the qualitative behavior of the order parameter and suggesting the existence of a spin ice ground state and pure Coulomb phase with zero ice rule violations at lower temperatures.

The Coulomb phase is further validated by the data’s correspondence to Debye-Hückel theory. At its core, this theory describes point charges that interact with one another via Coulomb’s law. These charges can emerge and disappear within the system via thermal activity or whenever energetically favorable. Because they are attracted to opposite charges and repelled by charges of the same sign, some portion of the charges spend more time near one another. This portion of the charges is referred to as the correlated charge density, leaving the rest as the uncorrelated charge density. Combining the fundamental physics of Poisson’s equation with charge density governed by Maxwell-Boltzmann statistics, these point charge densities are predicted to change as a function of temperature. The magnetic defects observed in our study obey these fundamental laws of thermodynamics and electrostatics through their agreement with the Debye-Hückel theory. The Coulomb interaction at the core of this theory further affirms the defects’ identity as emergent monopoles.

The current study focuses on field-free thermodynamics of emergent magnetic monopoles in extensively degenerate artificial square ice with height offsets between nanomagnets. Future research might take advantage of the real-space imaging aspect to explore field-dependent nonequilibrium response of emergent magnetic monopoles and establishing links to electro-diffusion theories (*35*). Furthermore, advances in nanofabrication techniques will allow researchers to fabricate similar artificial square ice patterns consisting of significantly smaller nanomagnet sizes and lower blocking temperatures (*17*) that will finally answer the long-standing question regarding the true spin ice ground state (*3*, *36*).

## MATERIALS AND METHODS

### Sample fabrication

The spin ice structures with height offsets were fabricated in two separate electron beam lithography exposure steps (fig. S2) for the plateau definition and then for the nanomagnets. First, gold marks were fabricated on a silicon substrate to align both electron beam exposure steps to the same set of marks. PMMA (polymethyl methacrylate) 950k C2 was spun at 2000 rpm to give a thickness of 170 nm. The pattern for the raised plateau was then exposed with a Vistec VB300 electron beam lithography tool at 100 kV in four separate quadrants of the silicon wafer to allow four different etch depths on the same substrate. The PMMA was then developed using a high-contrast cold development process consisting of 7:3 isopropyl alcohol (IPA):water solution ultrasonicated for 100 s. Cr (10 nm) was evaporated and lifted off to create the etch mask for the raised plateau. The silicon was etched in an Oxford Instruments reactive ion etcher with gas flow of 40-SCCM (standard cubic centimeter per minute) CHF_3_ and 8-SCCM SF_6_ at a pressure of 20 mtorr and a power of 50 W. Varying etch times gave the desired variation in etch depth. After stripping the Cr, PMMA was spun again on the substrate at 1000 rpm for the electron beam lithography exposure defining the nanomagnets. After exposure and cold development, a layer of 2.5-nm (*T*_B_ = 160 K) and 3-nm (*T*_B_ = 330 K) permalloy (Ni_80_Fe_20_) and a capping layer of 3 nm Al were evaporated and lifted off in dichloromethane. The exact values of the etched height offsets were determined using atomic force microscopy (AFM).

### X-ray PEEM

Measurements were performed using the cryogenic photoemission electron microscope PEEM3 at beamline 11.0.1 at the Advanced Light Source (*23*). Magnetic images were recorded by taking advantage of XMCD at the Fe L3-edge (*24*). The obtained contrast is a measure of the projection of the magnetization on the x-ray polarization vector so that nanomagnets with a magnetization parallel or antiparallel to the x-ray polarization appear either black or white. Nanomagnets with moments having ±45° and ±135° angles with respect to the incoming x-rays appear dark and bright, respectively. The silicon plateaus do not generate any disturbing background or shadow signal (*13*).

### Magnetic structure factors

The magnetic structure factor is calculated as

$$I(q)=\frac{1}{N}\sum_{i=1}^{N}\sum_{j=1}^{N}S_{i}^{⊥}\cdot S_{j}^{⊥}\text{exp}(iq\cdot r_{i,j})$$

where $S_{i}^{⊥}=S_{i}-(\hat{q}\cdot S_{i})\hat{q}$ is the component of the spin vector of each island, **S**_*i*_, perpendicular to the reciprocal space vector **q**,; the unit vector is given by $\hat{q}=q/||q||$; **r**_*i*,*j*_ is the vector from island *i* to *j*; and *N* is the total number of islands. This equation has the same form as in neutron scattering experiments and has previously been used to analyze artificial spin ice configurations.

### Pinch point analysis and correlation length

Pinch points are visible in the structure factor map of the *h* = 145 nm sample, for example, at the point (2,2). To analyze this quantitatively, we extracted a line scan of the structure factor through the pinch point, from the point (3/2,5/2) to the point (5/2,3/2), plotted in Fig. 2E. As in (*13*), we calculated the correlation length in the system, ξ, from a Lorentzian fit to the intensity profile

$$I(q)=A\frac{\xi^{-2}}{{\left(q-q_{0}\right)}^{2}+\xi^{-2}}+B$$

where *q* is the distance along the line scan, *q*_0_ is the location of the pinch point, and *A* and *B* are constants. Fitting the data of Fig. 2E, we find *A* = 4.75, *B* = 0.25, and ξ = 10.8*a* ± 0.1.

### Debye-Hückel analysis

The Debye-Hückel theory on plasmas (*28*) can be seen as a means to understand the liquid-gas transition and emergent magnetic monopole dynamics in pyrochlore spin ice. The basic idea is to describe the emergence of different categories of charges as carriers of energy. In this model of plasma, the charges emerge through Bjerrum ion pairing (*29*), splitting the population into correlated and uncorrelated charges. In the following analysis, the correlated monopoles are located adjacent another correlated monopole of opposite charge and homogenously distributed, while the distribution of the uncorrelated monopoles is completely uniform. The density of the correlated charges, number per total charge sites, is predicted as a function of uncorrelated charge density, temperature, and material parameters. Levin’s work on electrostatic correlations (*29*) outlines these predictions. One can de-unitize and combine equations for free energy, chemical potential, and charge densities (numbered 8, 13, 25, and 28) from Levin’s work (*29*) to find

$$\rho_{2}=\frac{C_{1}^{3}C_{2}}{128\pi T^{3}}\rho_{1}^{2}\text{exp}[\frac{1}{2\pi C_{2}}(\frac{1}{2\sqrt{\frac{T\rho_{1}}{C_{1}C_{2}}}}-\frac{1}{2\rho_{1}+2\sqrt{\frac{T\rho_{1}}{C_{1}C_{2}}}}-\frac{C_{1}C_{2}}{2T})]\int_{2}^{\frac{C_{1}}{4\pi T}}\frac{e^{u}}{u^{4}}du$$ (1)

where $C_{1}=\frac{4\pi Q^{2}}{k_{B}\epsilon a}$and $C_{2}=\frac{a^{3}}{V}$. *Q* is the charge, *a* is the lattice spacing, ρ_1_ is the number density of uncorrelated charges, and ρ_2_ is the number density of correlated charges. *T* is the temperature, *k*_B_ is the Boltzmann constant, ϵ is the permittivity of free space, and *V* is the volume the charges may occupy. In making the analogy to a magnetic system, ϵ is replaced with 1μ0.

This theory is scale free and therefore makes no direct prediction for the uncorrelated charge density that incites the correlated charge density. To find this value while making the least assumptions, we measured the total charge density. ρ_1_ may be written in terms of the total charge density, ρ = ρ_1_ + ρ_2_. This places ρ_2_ on the right hand side of Eq. 1, converting it into a self-consistency problem. This is solved using the method of relaxation. Implementing this process in whole requires experimentally observed total charge density and temperature, while *C*_1_ and *C*_2_ are left as fitting parameters. The result is a plasma theory generated prediction for the correlated charge density.

To experimentally define and measure magnetic charges, we use the so-called dumbbell model (*5*, *37*). Dipoles are the fundamental source of magnetic interaction, but not the only means of accounting for magnetic properties. To better understand frustrated systems, it is common to use the relationship between electric dipoles and charges as an analogy to define a magnetic charge. This approach converts dipole moment to two charges with opposite sign at a finite separation, creating the titular resemblance to a dumbbell. The magnitude of these charges *Q* is the magnetic moment divided by the dipole length *L*. This approximation is well established enough to serve as a description of the charged excitations in our system.

Correlated and uncorrelated charge densities can be determined through a calculation of the configurational energy and measurement of the total charge density. To do so, the square grid of dipoles is converted to a square grid of charges. The charges meeting at the corners of the square lattice are added together and approximated as one charge, *q*_*j*_ = *Q*_*j*_/2. The energy of nearest-neighbor interaction is mapped onto a $q_{j}^{2}$ term, while the long-range interactions take a Coulomb form

$$E=J_{nn}\sum_{j}q_{j}^{2}+J_{lr}\sum_{j<k}\frac{q_{j}q_{k}}{r_{jk}}$$

where *r*_*jk*_ is the distance between two charges in number of lattice parameters and all dimensional quantities are absorbed into *J*_*nn*_ and *J*_*lr*_, which can be calculated but will cancel in this analysis due to its geometric nature. The assertion that the system of charges acts like a plasma motivates the assumption of homogeneity. This homogeneity allows the correspondence of the dumbbell energy to the correlated and uncorrelated charge densities.

Each configuration has an associated total energy in this approximation. The long-range interactions in the uncorrelated charges are between an equal number of positive and negative charges at random distances. These interactions average to zero, making the uncorrelated charge energy contribution $E_{1}=J_{nn}\sum_{j}q_{j}^{2}$. Every charge will either be +1 or −1 in the theoretical plasma and *N*ρ_1_ charges are present, where *N* is the number of total charge sites. This simplifies the energy to *E*_1_ = *NJ*_*nn*_ρ_1_. The correlated charges share this first term, but their long-range interactions are nonzero due to a constant adjacent charge of opposite sign. All other long-range interactions will cancel, making the total correlated energy *E*_2_ = *NJ*_*nn*_ρ_2_ − *NJ*_*lr*_ρ_2_. The total energy is

$$\frac{E}{N}=J_{nn}(\rho_{1}+\rho_{2})-J_{lr}\rho_{2}$$

In reality, correlations need not take place at just neighboring charges, which introduces another set of energies from decreasingly correlated charges. Fortunately, any weakly correlated charges will have nearly the same energy as the uncorrelated charges and any close to completely correlated charges will approach the correlated charge energy. Measuring density in this way rather than imposing a direct classification of correlated and uncorrelated charges maintains the information of these partially correlated charges. One could correct this approximation in the future by including more correlated charge groups that constitute the system.

Both the total charge density, ρ = ρ_1_ + ρ_2_, and the energy as represented by the individual charges are immediately calculable from the data. Isolating ρ_2_ yields

$$\begin{array}{c} \rho_{2}=\frac{E}{NJ_{lr}}-\frac{J_{nn}}{J_{lr}}\rho \\ \rho_{2}=\frac{1}{N}\sum_{j<k}\frac{q_{j}q_{k}}{r_{jk}} \end{array}$$ (2)

As expected, this is a quantity purely derived from the arrangement of the charges. Once ρ_2_ is calculated, ρ_1_ is what remains of the total charge density.

These two models state independent expressions for the correlated charge density. Equation 1 corresponds to plasma theories of charge densities, while Eq. 2 relies on the verified dumbbell approximation and the experimental state of the spin ice system.

For this paper, the 145-nm offset array data at six temperatures were processed to yield charge densities as calculated by Eq. 2. SDs in these values were derived from real-time observations of thermal fluctuations for a period of approximately 20 min at each temperature. These densities were fitted to the Debye-Hückel theory as derived in Eq. 1 by scaling the magnetization of the dipoles within the expected range. *C*_1_ and *C*_2_ were left as fitting parameters, while the experimental data provided *T* and ρ.

### Crystallization order parameter

As another measure of charge ordering, a crystallization order parameter was calculated as defined by Brooks-Bartlett *et al*. (*34*)

$$M_{c}=\langle |\frac{1}{N}\sum_{i=1}^{N}q_{i}\Delta_{i}|\rangle$$ (3)

Here, the sum is taken over all *N* charge sites. The charge sites are checkered with Δ_*i*_ = ±1 so that a complete tiling of the charge sites with alternating plus and minus charges would result in *M*_c_ = 1. In contrast, a low-temperature spin ice has an order parameter of zero because most charge sites are vacant, and any emerging charges are independent of one another. This parameter quantifies global charge ordering, complementing the more local measure of correlated versus uncorrelated charges. The temperature dependence of this parameter may be compared to curves from Brooks-Bartlett *et al*. (*34*) as further confirmation of spin ice behavior and lack of charge crystallization.

The presence of either a crystallized or spin ice ground state can be determined from the chemical potential of charges in relationship to the energy necessary to separate charges. If the chemical potential is small, then charges will dominantly populate the system without dissociating, forming a crystal of charges. Higher chemical potential prevents charges from emerging until the temperature is high enough to immediately dissociate introduced charges. This latter description is required to facilitate the low density of monopoles characteristic of a spin ice (*5*).

The critical chemical potential equals the energy of a single charge interacting with a lattice otherwise filled by a charge crystal. We have already labeled the interaction energy with a single opposing charge as *J*_*lr*_. Units may be restored to these calculations by $J_{lr}=\frac{\mu_{0}m^{2}}{4\pi a^{3}}$ (*5*), where *m* is the magnetic moment of a single-spin island. Finding an entire crystal’s ionic energy in relationship to this interaction strength is a well-explored numerical problem. The solution simply multiplies this energy by an irrational number named the Madelung constant (*38*). Here, we seek the Madelung constant divided by two, as the energy belongs equally to the charge in question and every other charge in the system. For the square lattice, this constant is about 1.61554 (*39*), which makes the critical energy 0.80777*J*_*lr*_, translating to a critical chemical potential of μ_c_ = 0.80777*J*_*lr*_. De-unitizing in the same fashion as in the study of Brooks-Bartlett *et al*. (*34*), we write $\mu_{c}^{*}=\frac{\mu_{c}}{J_{lr}}=0.80777$. A system with a chemical potential below this will enter a charge crystal phase at low temperatures.

The chemical potential of the square ice can be found by flipping one spin in a charge-free state. Here, we assume that the energy of this spin is entirely found in dipole-dipole nearest-neighbor interactions. As seen in fig. S1, *J*_1_ is reduced by the height offset to approximately equal *J*_2_. In the dipole approximation

$$J_{2}=\frac{\mu_{0}m^{2}}{4\pi a^{3}}|{\hat{m}}_{1}\cdot {\hat{m}}_{2}-3({\hat{m}}_{1}\cdot \hat{r})({\hat{m}}_{2}\cdot \hat{r})|$$

Because the spins referred to, ${\hat{m}}_{1}$ and ${\hat{m}}_{2}$, and the unit vector joining them, $\hat{r}$,are all orthogonal, all dot products are one, so *J*_2_ = 2*J*_*lr*_.

A single spin in a charge-free state has four favorable interactions and two unfavorable interactions, making the net energy − 2*J*_1_. Flipping the spin to create two charges negates these interactions, resulting in an energy of 2*J*_1_. This cost the system 4*J*_1_ for two charges, making the chemical potential μ = 2*J*_1_ = 4*J*_*lr*_. This is de-unitized to μ* = 4, which is comfortably above the critical chemical potential. Charge crystallization is therefore not expected.

The order parameter was calculated for the 145-nm offset sample at 160 to 210 K (Fig. 4B). It briefly increased and then reached a stable value from 190 to 210 K, not exceeding *M*_*c*_ = 0.025. This behavior aligns with the spin ice state of a system found when the chemical potential lies above the critical value (*34*).

## Supplementary Material

http://advances.sciencemag.org/cgi/content/full/5/2/eaav6380/DC1

## SUPPLEMENTARY MATERIALS

Supplementary material for this article is available at http://advances.sciencemag.org/cgi/content/full/5/2/eaav6380/DC1 Fig. S1. SEM image of quasi–three-dimensional artificial spin ice. Fig. S2. Sample fabrication process. Fig. S3. Magnetic structure factors as a function of introduced height offset. Fig. S4. Illustrations of correlated and uncorrelated emergent magnetic monopoles. Fig. S5. Illustration of possible low-energy configurations, whether being a dilute gas of magnetic charges or a magnetic monopole crystalline ground state. Movie S1. XMCD image sequence of a thermally activated extensively degenerate artificial square ice (height offset = 145 nm) recorded at 190 K. Movie S2. Emergent magnetic monopole dynamics at 210 K (height offset = 145 nm).
