# Supplementary material for: Emergent magnetic monopole dynamics in macroscopically degenerate artificial spin ice
Source: Sci Adv. 2019 Feb 8;5(2):eaav6380. doi: 10.1126/sciadv.aav6380 (PMC6368442; doi:10.1126/sciadv.aav6380)
Supplement: http://advances.sciencemag.org/cgi/content/full/5/2/eaav6380/DC1 [file supp_5_2_eaav6380__index.html]

Science Advances | Science Advances

## Supplementary Materials

**The PDF file includes:**

- Fig. S1. SEM image of quasi–three-dimensional artificial spin ice.
- Fig. S2. Sample fabrication process.
- Fig. S3. Magnetic structure factors as a function of introduced height offset.
- Fig. S4. Illustrations of correlated and uncorrelated emergent magnetic monopoles.
- Fig. S5. Illustration of possible low-energy configurations, whether being a dilute gas of magnetic charges or a magnetic monopole crystalline ground state.
- Legends for movies S1 and S2

Download PDF

**Other Supplementary Material for this manuscript includes the following:**

- Movie S1 (.avi format). XMCD image sequence of a thermally activated extensively degenerate artificial square ice (height offset = 145 nm) recorded at 190 K.
- Movie S2 (.avi format). Emergent magnetic monopole dynamics at 210 K (height offset = 145 nm).

**Files in this Data Supplement:**

- Adobe PDF - aav6380\_SM.pdf
