# Supplementary material for: Emergent magnetic monopole dynamics in macroscopically degenerate artificial spin ice
Source: Sci Adv. 2019 Feb 8;5(2):eaav6380. doi: 10.1126/sciadv.aav6380 (PMC6368442; doi:10.1126/sciadv.aav6380)
Supplement: http://advances.sciencemag.org/cgi/content/full/5/2/eaav6380/DC1 [file aav6380_SM.pdf]

Science Advances
AAAS

advances.sciencemag.org/cgi/content/full/5/2/eaav6380/DC1

Supplementary Materials for

Emergent magnetic monopole dynamics in macroscopically degenerate artificial spin ice

Alan Farhan*, Michael Saccone, Charlotte F. Petersen, Scott Dhuey, Rajesh V. Chopdekar, Yen-Lin Huang, Noah Kent, Zuhuang Chen, Mikko J. Alava, Thomas Lippert, Andreas Scholl, Sebastiaan van Dijken

*Corresponding author. Email: alan.farhan@gmx.net

Published 8 February 2019, *Sci. Adv.* **5**, eaav6380 (2019)
DOI: 10.1126/sciadv.aav6380

The PDF file includes:

Fig. S1. SEM image of quasi–three-dimensional artificial spin ice.
Fig. S2. Sample fabrication process.
Fig. S3. Magnetic structure factors as a function of introduced height offset.
Fig. S4. Illustrations of correlated and uncorrelated emergent magnetic monopoles.
Fig. S5. Illustration of possible low-energy configurations, whether being a dilute gas of magnetic charges or a magnetic monopole crystalline ground state.
Legends for movies S1 and S2

Other Supplementary Material for this manuscript includes the following:

(available at advances.sciencemag.org/cgi/content/full/5/2/eaav6380/DC1)

Movie S1 (.avi format). XMCD image sequence of a thermally activated extensively degenerate artificial square ice (height offset = 145 nm) recorded at 190 K.
Movie S2 (.avi format). Emergent magnetic monopole dynamics at 210 K (height offset = 145 nm).

Supplementary Figures

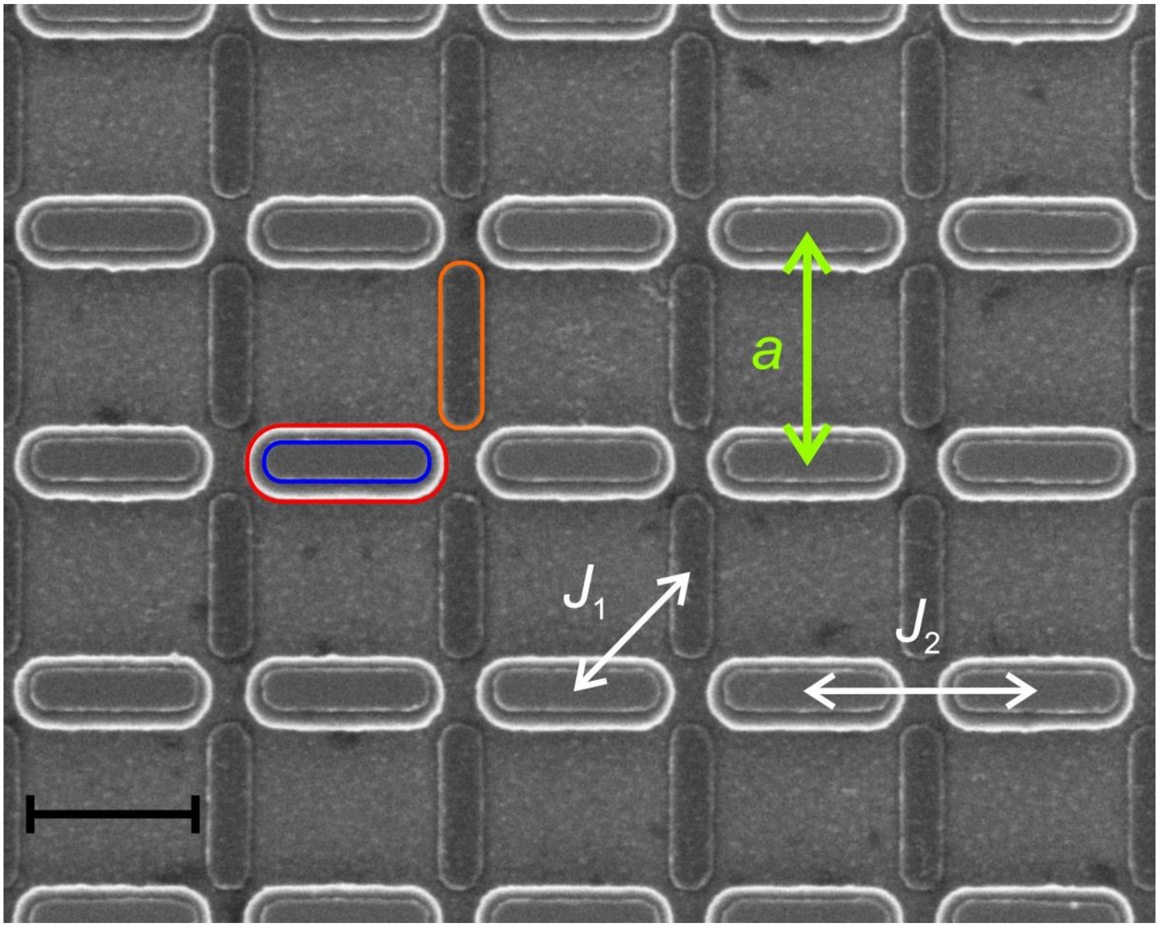

Fig. S1. SEM image of quasi–three-dimensional artificial spin ice. Scanning electron microscopy (SEM) image of thermally-activated artificial square ice array with an introduced height offset (h = 125 nm). The offset is introduced by growing Ising-type nanomagnets (length L = 400 nm, width W = 100 nm and thickness d = 2.5 or 3.0 nm) on a pre-etched silicon (100) substrate. One set of nanomagnets (blue-framed nanomagnet) are lithographically defined on top of etched plateaus (red-framed object), while the other set (orange-framed nanomagnet) is placed on the base of the substrate. With increasing height offset, the inter-nanomagnet coupling strength J_1 decreases, until a critical offset is achieved, where $J_1 = J_2$ and spin ice degeneracy is achieved. The black scale bar indicates a length of 400 nm.

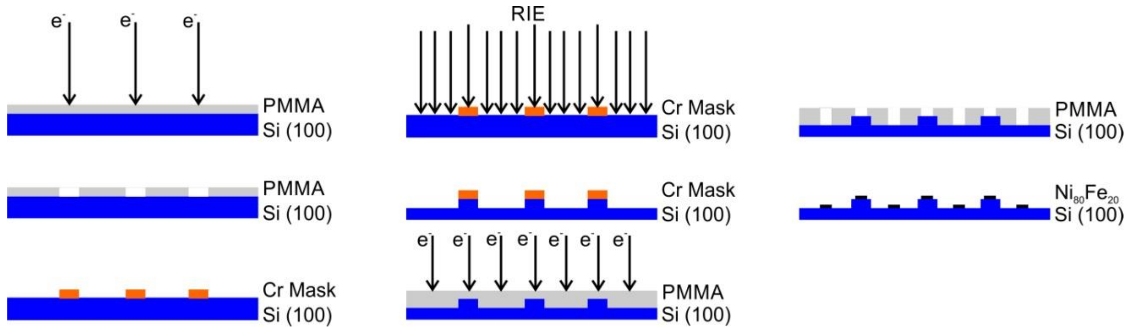

Fig. S2. Sample fabrication process. Schematic drawing of the nanofabrication process to create artificial square ice patterns with a controlled height offset. In a first lift-off assisted e-beam lithography step, a chromium mask is generated (left panel). Then, reactive ion etching (RIE) is used to etch the Silicon (100) substrate through the chromium mask, which is followed by a second e-beam exposure (middle panel), which generates the desired permalloy nanomagnets (right panel).

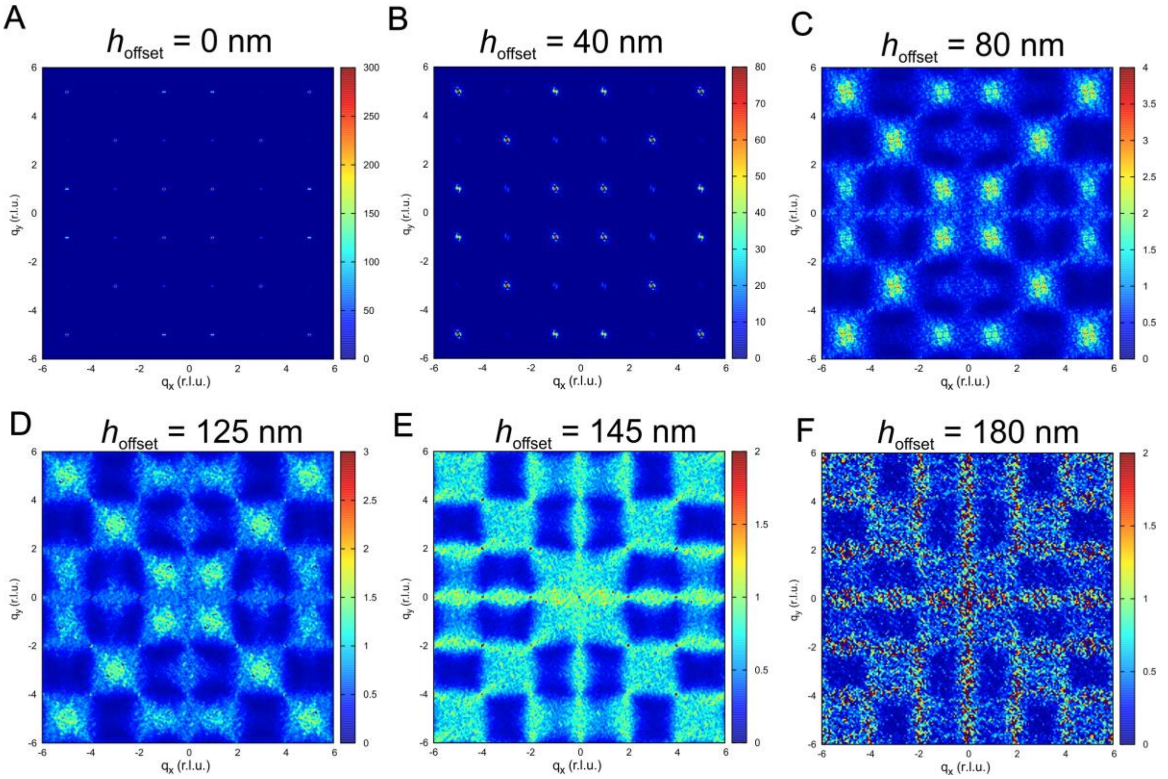

Fig. S3. Magnetic structure factors as a function of introduced height offset. Average magnetic structure factor generated from magnetic moment configurations imaged after thermal annealing on artificial square ice with height offsets of **A,** h = 0 nm, **B,** h = 40 nm, **c,** h = 80 nm, **D,** h = 125 nm, **E,** h = 145 nm and **F,** h = 180 nm. The colored scale bar is a measure of the intensity at a given point (q_x, q_y) in reciprocal space.

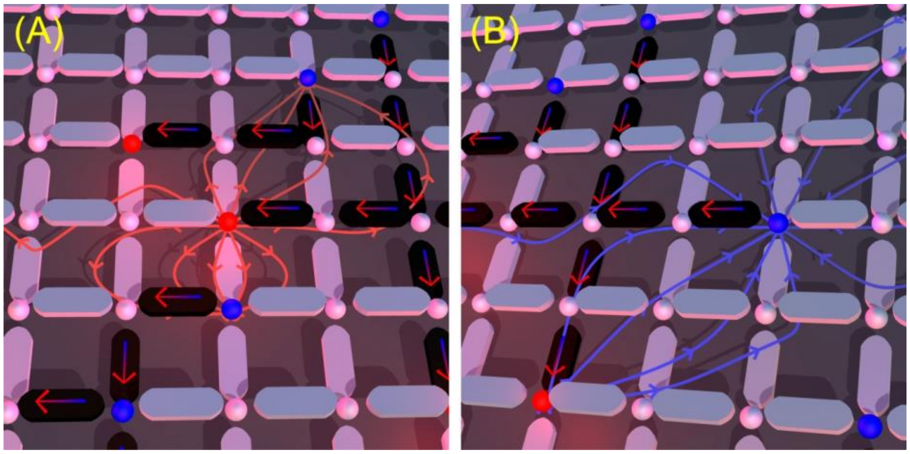

Fig. S4. Illustrations of correlated and uncorrelated emergent magnetic monopoles. A, Illustration of an artificial spin ice with a correlated monopole. To illustrate similarity to a magnetic dipole, field lines are drawn outward from the positive $+2q$ charge (red sphere) to the correlated negative $-2q$ charges (blue spheres). Although other charges interact with the correlated charge, its proximity to its adjacent negative charge ensures that the two will dominantly interact with one another due to energetic stability. In contrast with the uncorrelated charge, most of the field lines converge nearby. **B,** Schematic of converging field lines on an uncorrelated charge. The uncorrelated charge interacts with surrounding positive and negative charges. In isolation, the negative charge would tend to correlate with the nearby positive charge. The other randomly distributed charges screen this interaction, which can be seen in the field lines appearing more like those of a point charge and less like those of a dipole. This corresponds with this uncorrelated charge's long-range energy averaging to zero.

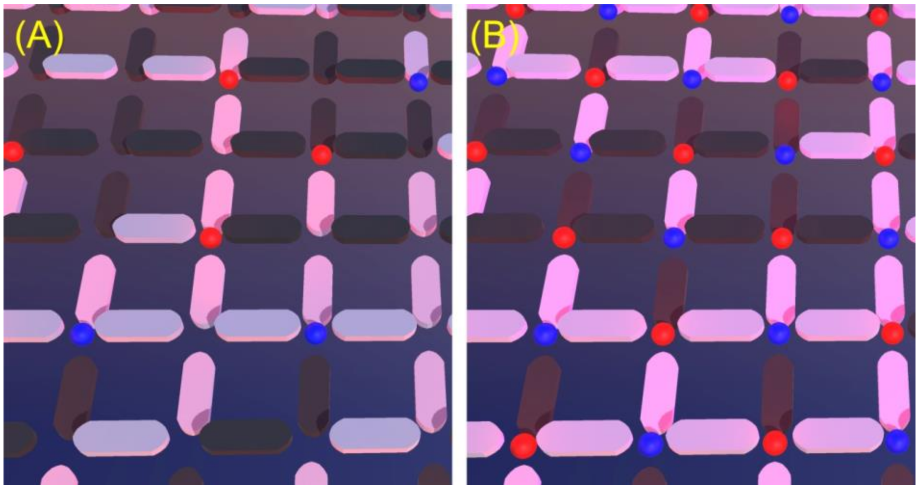

Fig. S5. Illustration of possible low-energy configurations, whether being a dilute gas of magnetic charges or a magnetic monopole crystalline ground state. Illustrations of possible low energy configurations. The neutral charge sites are left blank and charges are colored as in fig. S4. **A,** Dilute gas of magnetic charges emerging from a spin glass ground state. There is no global ordering as is the case in our experiments. The

charges are free to separate due to low Coloumb attraction compared to the chemical potential. **B,** Magnetic monopole crystalline ground state. The charges freeze in one of two global configurations.

Supplementary Movie Captions

Movie S1. XMCD image sequence of a thermally activated extensively degenerate artificial square ice (height offset = 145 nm) recorded at 190 K.

Movie S2. Emergent magnetic monopole dynamics at 210 K (height offset = 145 nm).
